# An electrochemical method for sensitive and rapid detection of FAM134B protein in colon cancer samples

**DOI:** 10.1038/s41598-017-00206-8

**Published:** 2017-03-09

**Authors:** Farhadul Islam, Md Hakimul Haque, Sharda Yadav, Md Nazmul Islam, Vinod Gopalan, Nam-Trung Nguyen, Alfred K. Lam, Muhammad J. A. Shiddiky

**Affiliations:** 1 Cancer Molecular Pathology Laboratory in School of Medicine, Menzies Health Institute Queensland, Griffith University, Gold Coast Campus, Australia; 2 School of Natural Sciences, Griffith University, Nathan Campus, QLD 4111 Australia; 3 Queensland Micro and Nanotechnology Centre, Griffith University, Nathan Campus, QLD 4111 Australia

## Abstract

Despite the excellent diagnostic applications of the current conventional immunoassay methods such as ELISA, immunostaining and Western blot for FAM134B detection, they are laborious, expensive and required a long turnaround time. Here, we report an electrochemical approach for rapid, sensitive, and specific detection of FAM134B protein in biological (colon cancer cell extracts) and clinical (serum) samples. The approach utilises a differential pulse voltammetry (DPV) in the presence of the [Fe(CN)_6_]^3−/4−^ redox system to quantify the FAM134B protein in a two-step strategy that involves (*i*) initial attachment of FAM134B antibody on the surface of extravidin-modified screen-printed carbon electrode, and (*ii*) subsequent detection of FAM134B protein present in the biological/clinical samples. The assay system was able to detect FAM134B protein at a concentration down to 10 pg μL^−1^ in phosphate buffered saline (pH 7.4) with a good inter-assay reproducibility (% RSD = <8.64, *n* = 3). We found excellent sensitivity and specificity for the analysis of FAM134B protein in a panel of colon cancer cell lines and serum samples. Finally, the assay was further validated with ELISA method. We believe that our assay could potentially lead a low-cost alternative to conventional immunological assays for target antigens analysis in point-of-care applications.

## Introduction

Family with sequence similarity 134, member B (FAM134B), also called JK1, is a protein involved in a number of chronic diseases including neuronal disorders, vascular dementia, allergic rhinitis and cancers^1–4^. FAM134B protein is reported to function as a tumour suppressor and its’ changes in expression was associated with different pathological stages in patients with colorectal carcinomas^5^. Additionally, FAM134B expression was correlated with poor survival in colorectal cancer patients^6–8^. Thus, an accurate and specific detection of this protein with a simple method could be used as a liquid biopsy for monitoring its role in the progression or recurrence of colorectal cancer^9–13^.

Current conventional methods such as enzyme linked immuno-sorbent assay (ELISA), immunostaining, western blot has excellent analytical performance for FAM134B detection, but these conventional detection techniques require large amounts of sample and extensive technical steps. They also require high running/maintenance cost, cumbersome and laboratory-based procedures, technical experts, long assay protocol and complicated data analysis, which limits their application in inexpensive and fast point-of-care analysis^14, 15^. Therefore, there is a demand for new assays that can minimize the challenges of conventional detection methods, and will provide non-invasive and quick detection of FAM134B protein.

Electrochemical readout is suitable for clinical settings due to their high sensitivity and specificity, cost-effective measurements, and compatibility with the miniaturization (*i.e.*, amenable with the point-of-care devices)^10, 16, 17^. A practical advantage of electrochemical detection could have future implications in translating to cheap assays using single-use screen-printed electrodes, which is an ideal tool due to their low cost, disposability and design flexibility as compared to traditional electrode materials^18, 19^. Electrochemical analysis of FAM134B protein using single-use disposable electrode has yet to be demonstrated. Herein, we report a proof-of-concept electrochemical approach to address the limitations of conventional detection approaches for analyzing FAM134B protein in biological and clinical samples from colorectal cancer patients.

Extravidin-modified screen-printed electrode was first functionalized with biotinylated anti-FAM134B antibody. The electrodes were then incubated with the protein of interest, FAM134B. Differential pulse voltammetry (DPV), in presence of the [Fe(CN)_6_]^4−/3−^ redox system, was used to monitor the Faradaic currents generated in each step of this sensing layers^20–22^. The addition of subsequent antibody and protein layers on the electrode surface blocks the [Fe(CN)_6_]^4−/3−^ redox system from accessing the surface quite effectively, which results in a decrease in DPV current response. Therefore, the decrease of current generated by the [Fe(CN)_6_]^4−/3−^ system after FAM134B protein binding should have a clear correlation with the concentration of proteins. We found that the assay system is relatively specific, sensitive, rapid, stable and can be used with unprocessed complex biological (whole cell extracts) and clinical (serum) samples. The results are consistent with that of standard methods such as ELISA and immunostaining. The additional benefit of this assay system is relatively faster, simpler, easier to execute and most importantly cost effective.

## Results and Discussion

### Assay Principle

**Figure 1:**
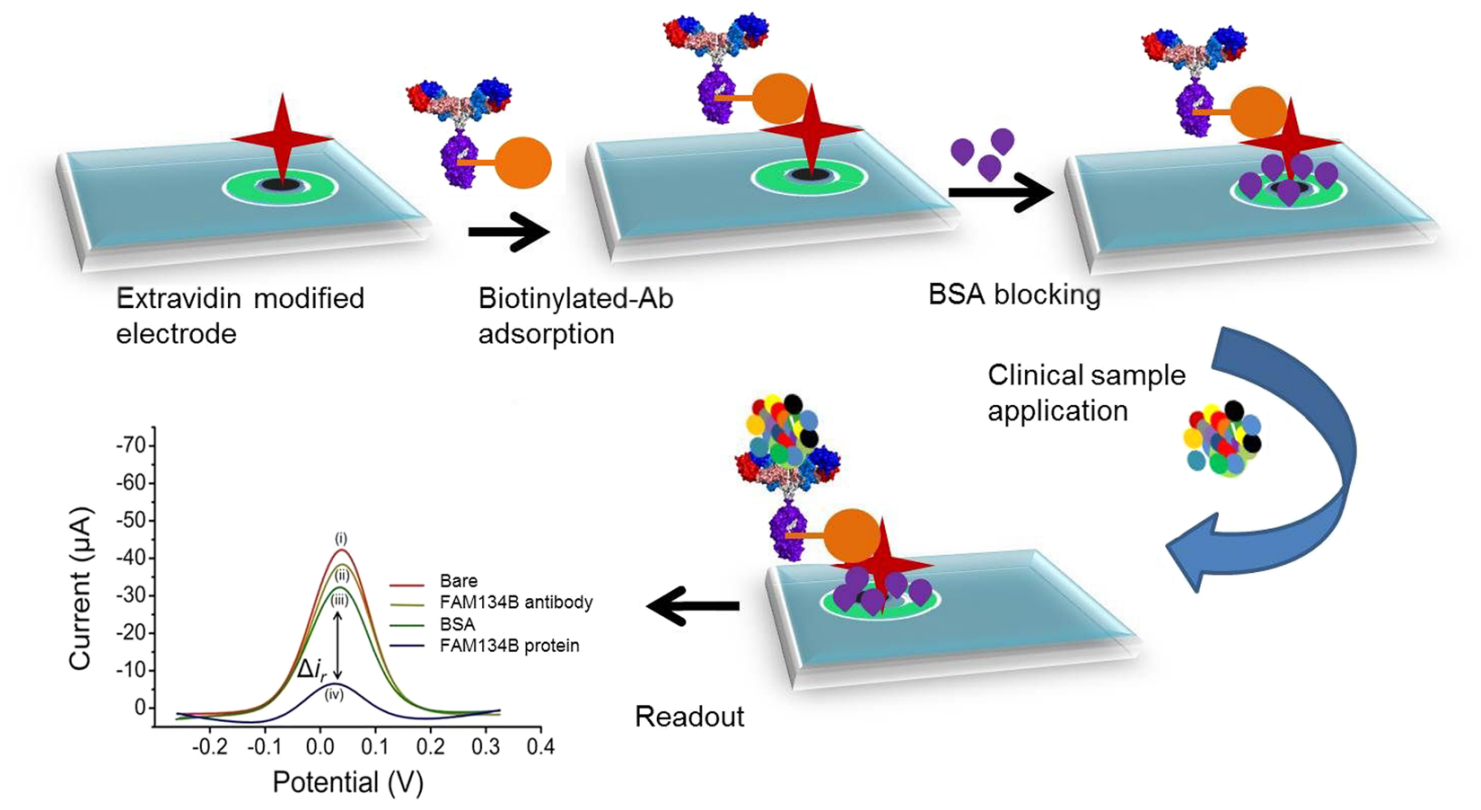
Approach for the detection of FAM134B protein in colon cancer. Schematic showing the steps involved in the fabrication of electrochemical assay for the detection of FAM134B protein. FAM134B proteins extracted from colon cancer cell lysates and serum samples complexed with surface attached-biotinylated FAM134B antibody which hinders the interfacial electron transfer reaction of [Fe(CN)_6_]^3−/4−^. Inset, the DPV current generated at the (*i*) unmodified and (*ii*-*iv*) modified electrodes. Δ*i*r is the difference between relative DPV signals obtained at antibody/BSA- and antibody/BSA/antigen-modified electrodes.

Figure 1 illustrated our current approach for the detection of FAM134B protein in complex biological/clinical samples using extravidin-modified screen-printed carbon electrodes. Briefly, biotinylated anti-rabbit monoclonal FAM134B antibody was first captured onto the extravidin-coated electrode. As extravidin provides stable high affinity surface for a large amount of biotinylated molecules in comparison to the streptavidin modified surface, this interaction allowed binding of FAM134B antibody to the electrode surface^23, 24^. The specificity of the antibody used in this study was checked using Western blot analysis (see Figure S1). The BSA step was then followed by the FAM134B protein or biological/clinical sample incubation step. The target FAM134B biomarker was bound to the surface-immobilized FAM134B antibodies. In this method, the stepwise attachment of biotinylated-FAM134B antibody, BSA and FAM134B protein on the extravidin-coated electrode were tested by monitoring the Faradaic current (*i.e.*, in this case the DPV current) generated by the [Fe(CN)_6_]^3−/4−^ system present in the electrolyte solution. Stepwise addition of the assay components on the sensor surface, blocks the [Fe(CN)_6_]^3−/4−^ redox system from accessing the electrode surface, which in turn decreased the DPV current signal^13, 21, 22, 25^. For example, compared to the bare electrode (Fig. 1(i)), addition of biotinylated-FAM134B antibody showed a reduced level of DPV response (Fig. 1(ii)). Subsequent surface blocking by BSA led to an additional reduction of DPV signal (Fig. 1(iii)). Further reduction of DPV current signal was observed after the addition of FAM134B protein to the sensor surface (Fig. 1(iv)). The decrease of current signal with the stepwise addition of individual biomolecule clearly indicates a successful build-up of a biosensor assembly on the extravidin-modified screen-printed electrode.

### Optimization of the Experimental Parameters

The applicability of this sensing system is limited by the amount of the biomolecules immobilized in the building of the recognition layer (in this case, amount of antibody and BSA on the extravidin-modified screen printed electrode). This is because there is only a finite amount of effective surface and having the large amount of antibody and/or BSA could completely block the [Fe(CN)_6_]^3−/4−^ redox system from accessing the electrode surface (*i.e.*, saturation of the effective surface), leading to a situation where extent of target FAM134B protein would make indiscernible signal difference. Therefore, to achieve the optimal analytical performance of the biosensor (*i.e.*, relatively larger Δ*i*_r_ value for a wide range of FAM134B concentration), we optimized the binding conditions (*i.e.*, concentration and incubation time) for FAM134B antibody and BSA. To obtain the optimal concentration of FAM134B antibody, we tested its incubation at 1, 10, 50 and 100 ng μL^−1^ on the extravidin-modified electrode surface for 20 min with 100 pg μL^−1^ of FAM134B protein.

**Figure 2:**
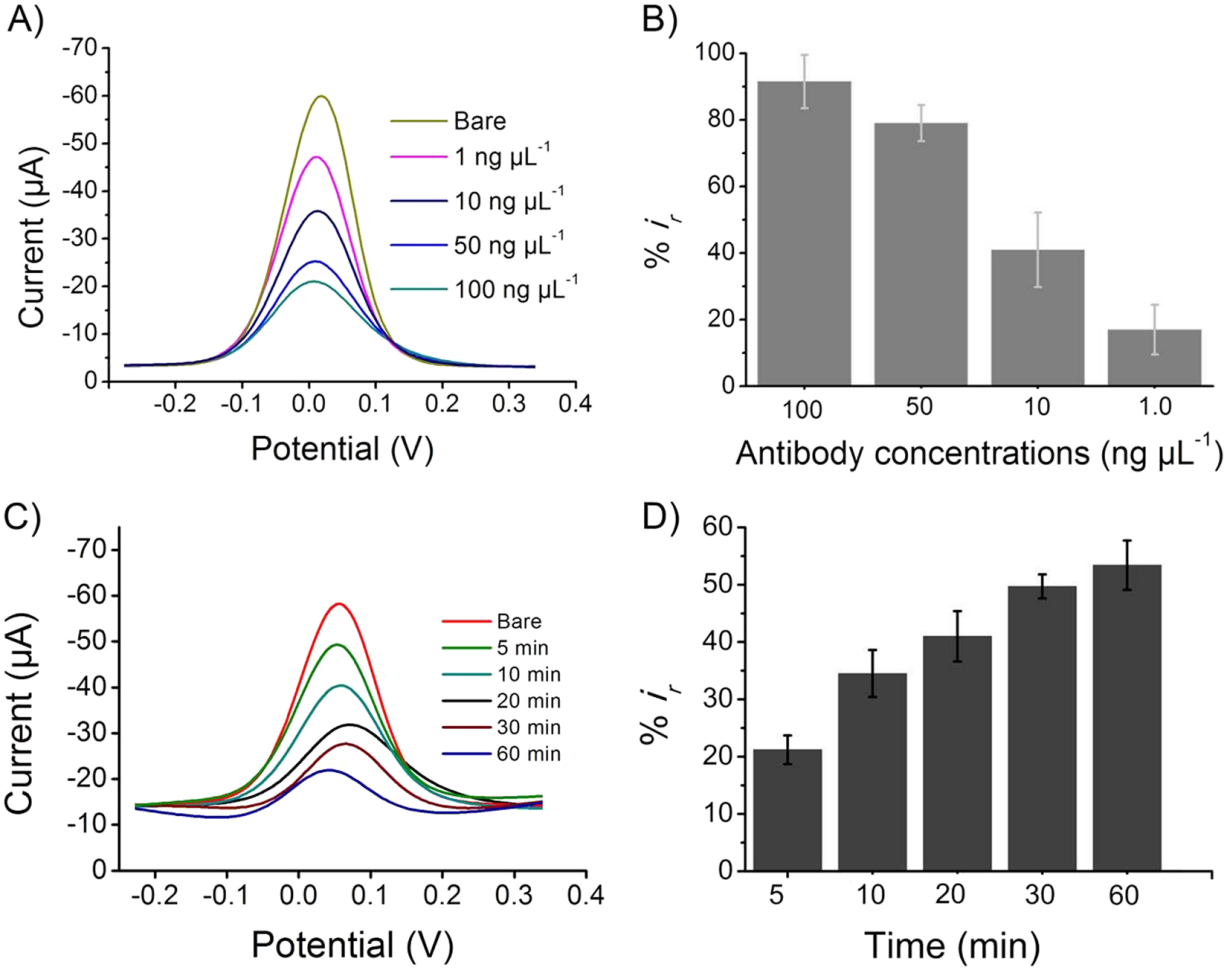
Optimization of the experimental conditions. Typical differential pulse voltammetry signals (**A**,**C**) and corresponding relative current changes (**B**,**D**) after the adsorption of FAM134B antibody on the extravidin-modified screen-printed electrode. (**A**) the designated concentrations (1–100 ng µL^−1^) of FAM134B antibody was immobilized for 20 min. (**C**) 10 ng µL^−1^ of FAM134B antibody was immobilized for the designated times (5–60 min). Each data point represents the average of the three separate trials (*n* = 3) and error bars represents the standard deviation of measurements within experiments.

Figure 2A shows that the level of immobilized FAM134B antibody effectively reduces the DPV current in comparison to that of the bare electrode. We noted that 1.0 ng μL^−1^ of FAM134B antibody generated a much lower level of current reduction for 100 pg µL^−1^ protein with respect to the bare electrode (%*i*_*r*,*FAM134B antigen*_ = 17%), whereas 50 ng μL^−1^ and 100 ng μL^−1^ FAM134B antibody produced much higher current reduction (%*i*_*r*,*FAM134B antigen*_ = 79 and 91.5% respectively) (Fig. 2A,B). Clearly, high concentration of FAM134B antibody resulted an electrode surface with high level of surface-bound higher density antibody that would induce high steric barrier for the [Fe(CN)_6_]^3−/4−^ redox system from accessing the electrode surface (a condition where higher concatenation of FAM134B protein would make indiscernible signal difference). In the case of lower concentration of FAM134B antibody, the detection sensitivity of the assay would affect significantly (i.e., insufficient surface-bound antibody for the target protein attachment). The sensor fabricated using 10 ng μL^−1^ of FAM134B antibody for 20 min incubation resulted a %*i*_*r*,*FAM134B antigen*_ = 41% current reduction in comparison to the bare electrode. This data clearly indicates that 10 ng μL^−1^ of FAM134B antibody concentration is high enough for target attachment, while still provide space to access [Fe(CN)_6_]^3−/4−^ system for monitoring subsequent FAM134B protein attachment steps. Therefore, 10 ng μL^−1^ was selected as optimal concentration of FAM134B antibody.

Incubation time is another important factor affecting the analytical performances of our assay^13^. We optimized the incubation time (5, 10, 20, 30, 60 min) for 10 ng μL^−1^ of FAM134B antibody attachment by measuring the relative current changes for 100 pg μL^−1^ of target FAM134B protein. The relative current change was found to be increased with the increasing of incubation time (Fig. 2B). Approximately 50% DPV current deduction (%*i*_*r*,*FAM134B antigen*_ = 50%) was noted with 100 pg μL^−1^ protein concentration after 30 min of incubation. Further longer incubation time did not cause in significant relative current changes (Fig. 2C,D) and therefore 30 min was selected as optimal incubation time for FAM134B antibody.

To prevent non-specific adsorption of proteins and other biological moieties on the antibody-bound electrodes, it is necessary to block the electrode with non-specific filler like BSA. To this end, we optimized the concentration of BSA (0.01 to 5%) at 20 min of incubation time. As shown in Figure S2, approximately 40% DPV current deduction (%*i*_*r*,*antibody/BSA*_ = 40%) was found for 0.1% BSA for 20 min of incubation. At >0.1% BSA concentration, a saturation of the %*i*_*r*,*antibody/BSA*_ was achieved and hence 0.1% BSA was selected as optimised concentration.

### Control Experiments and Analytical Performance of the Assay

**Figure 3:**
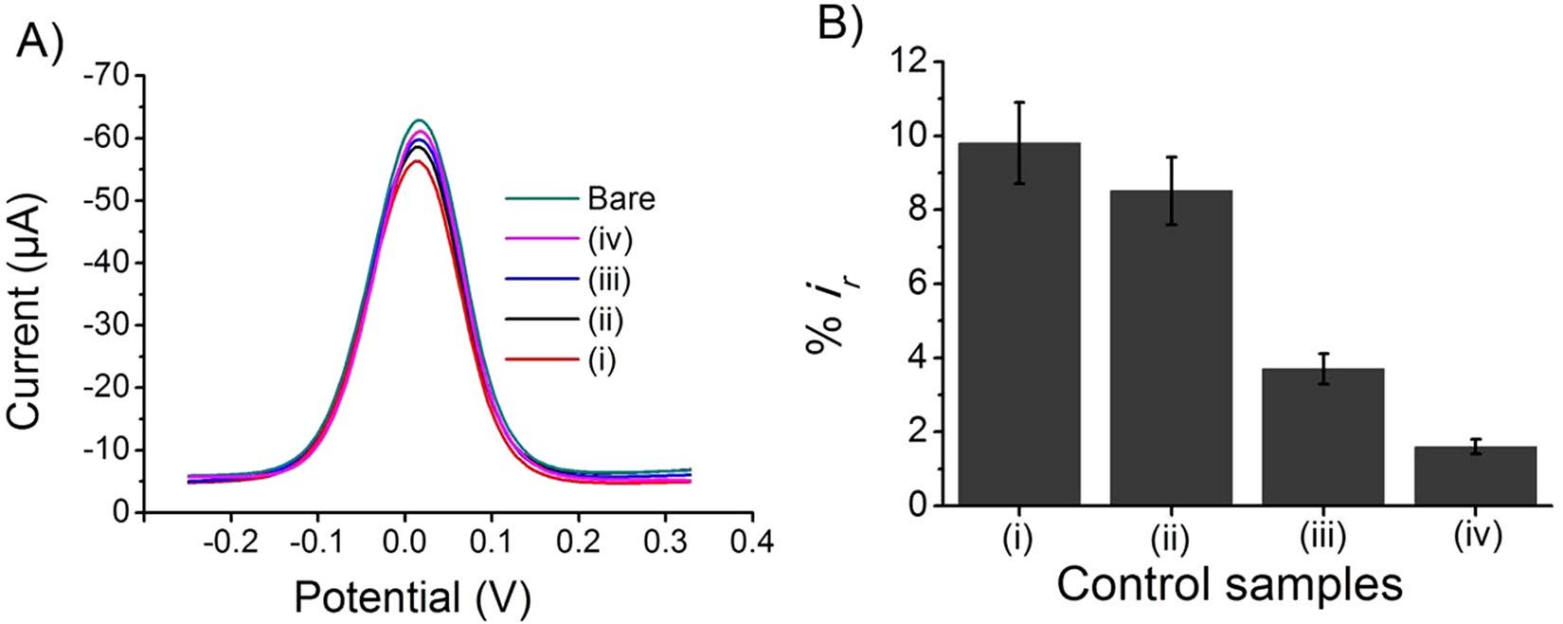
Control experiments. Typical differential pulse voltammetry signals (**A**) and corresponding relative current changes (**B**) obtained at the electrodes modified with (*i*) HER-2 antibody + FAM134B protein, (*ii*) FAM134B antibody + HER-2 protein, and (iii, iv) FAM134B proteins knockout SW48 (out 1) and SW480 (out 2). Each data point represents the average of the three separate trials (*n* = 3) and error bars represents the standard deviation of measurements within experiments.

To examine the level of nonspecific and cross reactive interactions involved in our assay, we carried out a number of control studies under optimised conditions. To assess these possible interactions, we used electrode captured with (*i*) nonspecific HER2 antibody reacted with FAM134B antigen, (*ii*) FAM134B antibody interacted with non-target HER2 protein and (*iii*) FAM134B immuno-precipitate out sample (see Figure S3). We found 9.80% relative current change when electrodes were functionalised with nonspecific HER2 antibody and incubated with FAM134B protein (nonspecific antibody-antigen reaction) at a concentration of 100 pg μL^−1^ (Fig. 3(i)). In case of HER2 protein (100 pg μL^−1^) and FAM134B antibody interaction (nonspecific antigen-antibody reaction), we observed only 8.51% of current reduction when compared to the bare electrode (Fig. 3(ii)). Similarly, FAM134B protein knockout SW48 and SW480 cancer cell extracts samples generated only 3.7 and 1.6% reduction of relative DPV current signals respectively (Fig. 3(iii–iv)). These results indicated that the nonspecific and cross reactive interactions in our assay are minimal.

The reproducibility of our detection system was tested for every experiment in triplicates. The relative standard deviation (% RSD) for FAM134B protein (100 pg μL^−1^) was 7.13% (*n* = 3), while in control study, for example HER2 protein detection with FAM134B antibody % RSD was found to be 6.6% (*n* = 3). A %RSD of 9.32% (*n* = 3) was noted for FAM134B protein detection with HER2 antibody, whereas 8.1% (*n* = 3) was found in case of FAM134B protein out samples analysis. The inter-assay reproducibility was found to be <8.64% (%RSD, *n* = 3).

**Figure 4:**
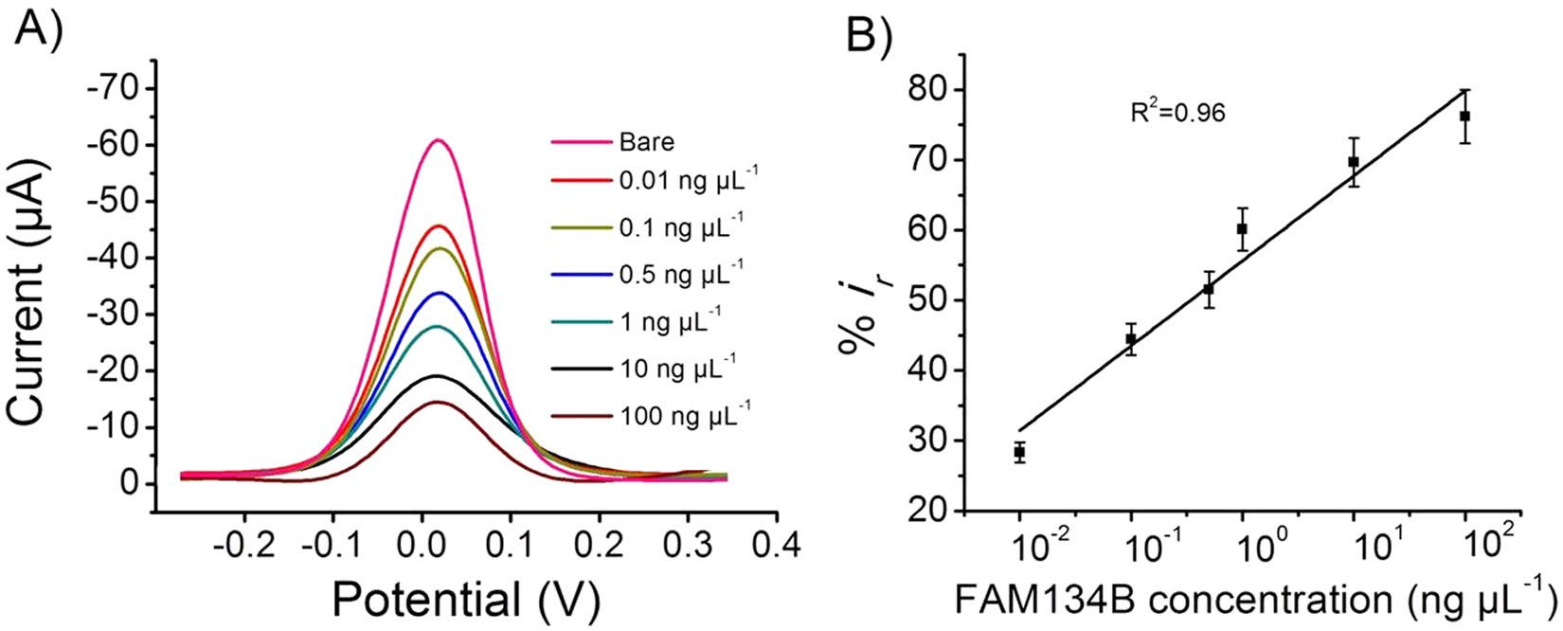
Assay performance. (**A**) Change of the DPV responses at the immunosensing surface after incubation with designated concentrations of FAM134B protein. (**B**) Corresponding relative current change-concentration curve. Each data point represents the average of the three separate trials (*n* = 3) and error bars represents the standard deviation of measurements within experiments.

To determine the limit of detection (LOD) and the sensitivity of the assay, designated concentration of standard FAM134B protein (0.01–100 ng μL^−1^) was spiked in PBS and quantified them by our assay system. We found a linear increment of relative current changes with the increase of FAM134B protein concentrations under optimized conditions in the dynamic range of 0.01 to 100 ng μL^−1^ with a correlation coefficient (*r* ^2^) of 0.96 (Fig. 4). This dynamic range of detection indicated that our method could potentially be applicable for detection and analyzing FAM134B protein in complex biological matrixes with various concentration of FAM134B antigen. The linear regression equation was *y* (*i*_*r*_%) = 55.64 logC + 12.10. The detection limit was found to be 10 pg μL^−1^ with a corresponding signal-to-noise ratio of 3 (Figs 4 and S4). Saturation of the electrode surface begins to occur at >100 ng μL^−1^ FAM134B protein concentrations. This is because high accumulation of proteins on the electrode completely blocks the [Fe(CN)_6_]^3−/4−^ redox system from accessing the electrode surface. We then compared the analytical performance of our assay system with that of the standard ELISA method and noted that our assay system was comparable to the ELISA in regards of sensitivity and LOD (see Figures S5A and B).

### Profiling of FAM134B Expression in Cancer Cell Lines

**Figure 5:**
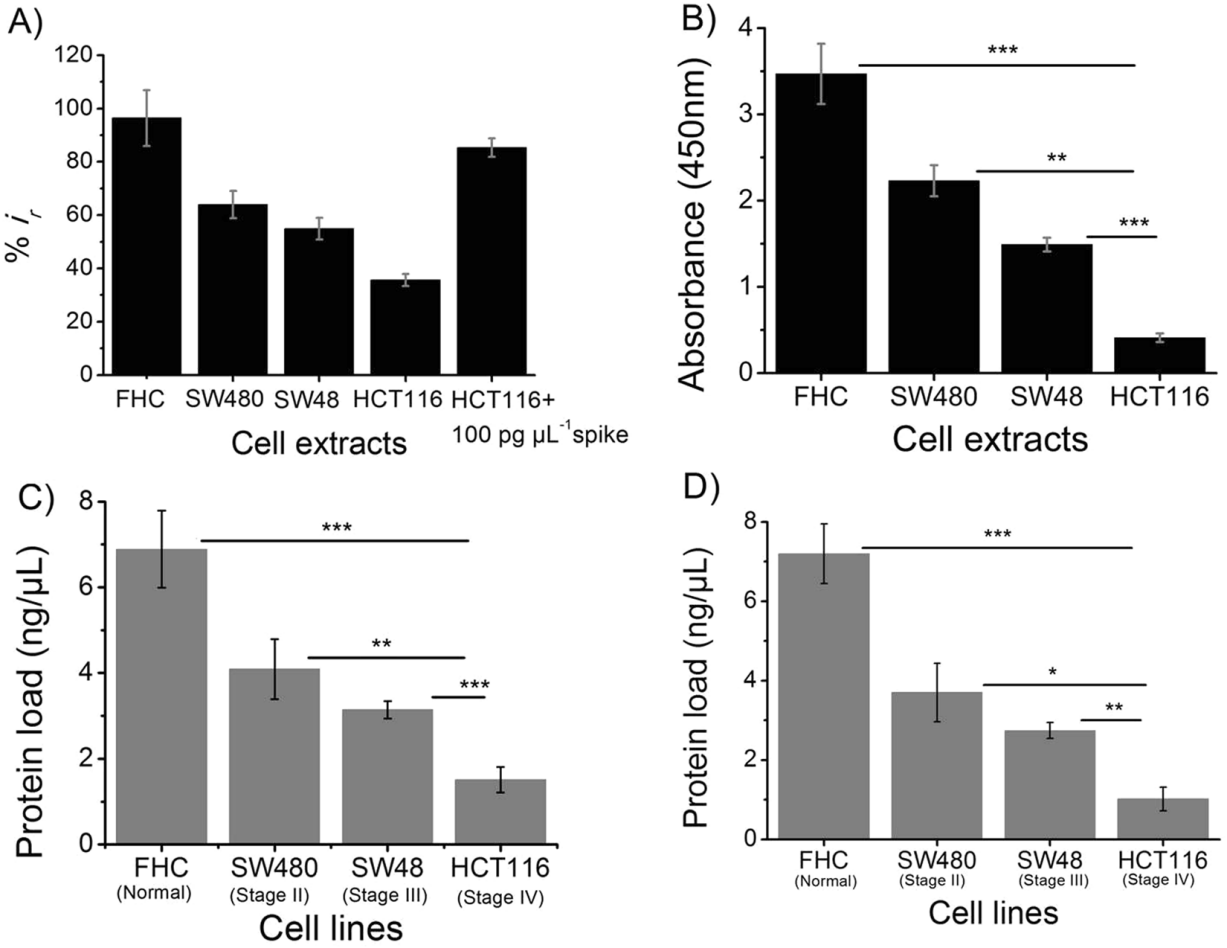
Profiling of FAM134B expression in cancer cell lines. Comparison of relative (**A**) current change and (**B**) absorbance response obtained for 7 µL of FHC, SW480, SW48, and HCT116 cell extracts. The relative current change obtained for the 7 µL HCT116 cell extract spiked with 100 pg µL^−1^ FAM134B protein is also included in (**A**). FAM134B protein concentrations (in ng μL^−1^) in FHC, SW480, SW48, and HCT116 cell extracts were measured using the standard curves obtained from (**C**) electrochemical and (**D**) ELISA measurements (see Figs 4 and S5B). Each data point represents the average of the three separate trials (*n* = 3) and error bars represents the standard deviation of measurements within experiments. Statistical significance were determined by pairwise comparison between two experimental groups using student's *t*-test *p < 0.05, **p < 0.01 and ***p < 0.001 when compared among/between the experimental groups.

To investigate the analytical reliability and assay performance of our method for biological sample analysis, we used whole cell lysates extracted from a panel of colon cancer (SW-480, SW-48 and HCT116), and non-neoplastic colon epithelium (FHC) cell lines. These cell lines were established from different pathological stages of colon cancer (SW-480, stage II colonic adenocarcinoma; SW-48, stage III colonic adenocarcinoma; HCT116, stage IV colonic carcinoma, and FHC, non-cancer colon epithelium)^26–30^. Using the standard curve generated from our detection system, we determined the protein concentration in each cell line. FAM134B protein load in cancer cell, originated from the higher cancer stages was significantly lower when compared to that of non-cancer cells (Fig. 5A,C and Figure S6A,B). For example, in normal colon epithelial cell (FHC), we found 96.4% reduction of relative DPV signal (%*i*_*r*_) whereas in stage IV colon cancer cell (HCT116) we observed only 35% reduction of current signal. In case of stage II (SW480) or stage III (SW48) colon cancer, we noted 63.92 and 54.95% of reduction of relative DPV response respectively (Fig. 5A). Notably, DPV voltammograms collected from cell lysate are very similar to the ones collected from straight spiked samples in PBS. A similar level of DPV voltammograms from the biological samples have previously been reported^20, 21, 25^. These data indicated that our assay is capable of quantifying differential expression pattern of FAM134B protein in different stages of colon cancer, and the difference is statistically significant. Differential expression of FAM134B protein in different stages of colon cancer was further confirmed with the standard ELISA method (Fig. 5B) and immunofluorescence microscopic analysis (see Figure S6B). These results are also in good accordance to our previous study, where we used conventional analytical methods such as qPCR, Western blot, immuno-staining for analyzing FAM134B expression. We reported that FAM134B expression was reduced in colon cancer with the advancement of the diseases stages^5^. This implied that our proposed method has analytical reliability and the performances are comparable to the standard methods.

We then quantified the level of FAM134B expression in different stages of colon cancer with our assay platform using calibration curve constructed from known amount of FAM134B protein. We found maximum protein load (6.89 ng μL^−1^) in non-cancer epithelial colon (FHC) cells. On the other hand, only 1.51 ng μL^−1^ (minimum) protein load was observed in the most advanced stage (stage IV) of colon cancer (HCT116) cells. Stage II (SW480) and stage III (SW48) colon cancer showed 4.09 and 3.14 ng μL^−1^ of FAM134B protein respectively (Fig. 5C). We also determined the protein concentration of four different cell lines using ELISA and observed that the results were comparable with our current method (Fig. 5D). This data indicates that our method is capable of measuring differential quantitative presence of FAM134B protein in cancer cell lines originated from different stages of colon cancer.

Finally, to confirm the assay specificity and full applicability of our system, we spiked known amount of FAM134B protein (100 pg μL^−1^) into the whole protein extract obtained from HCT116 cell line. We found a significant reduction of DPV signal (~85%) in spiked samples on top of the corresponding non-spiked sample (35%). The percentage changed (~50%) due to the addition of FAM134B in complex biological sample, indicating that our assay method is highly effective for specific and selective detection of FAM134B in the presence of large number of other proteins (Fig. 5A). Thus, our assay complex can challenge the complex biological samples, and is capable of detecting the target of interest in presence of competitive other proteins. Therefore, we believe that the use of extravidin-biotin-antibody affinity could potentially be an effective alternative to the conventional immunoassays, which in turn can provide easy and cost effective detection of immunotargets in clinical settings.

### Detection of FAM134B from Patients with Colorectal Cancer

**Figure 6:**
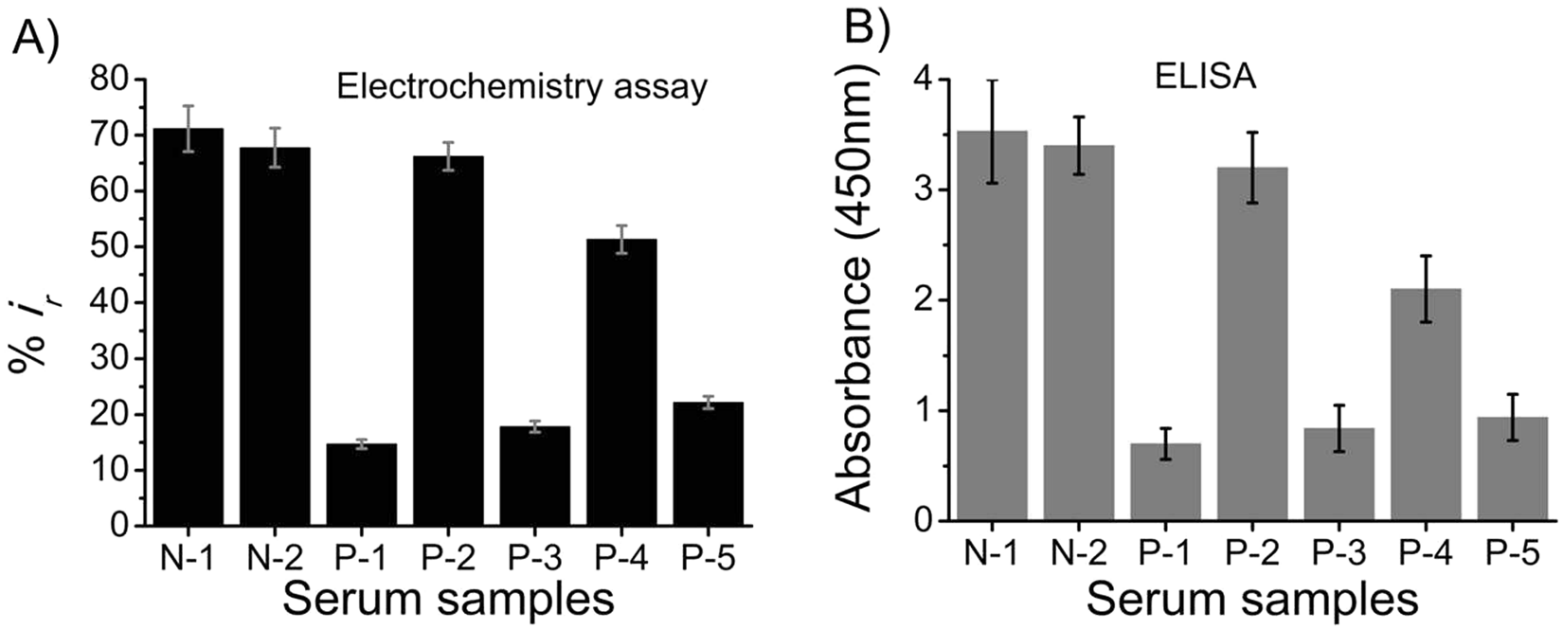
Clinical sample analysis. Comparison of relative (**A**) current change and (**B**) absorbance response between colon cancer patients (P1-P5) and healthy individuals (N1, N2). Each data point represents the average of the three separate trials (*n* = 3) and error bars represents the standard deviation of measurements within experiments.

The FAM134B protein was located in the nuclei of the tumour cells at both cellular and tissue levels. It was first time quantified in oesophageal cancer tissues by Tang *et al.* in 2007 using a RT-PCR^31^. The subsequent immunohistochemistry studies by our group has also reported the FAM134B expression changes in pre-cancerous (adenoma) and cancerous tissues samples from colorectum^6, 7^. In colorectal cancer, lower levels of FAM134B protein expression were associated with younger age (p = 0.032), larger tumour size (p = 0.004), advanced cancer stages (p = 0.016) and higher rates of cancer recurrence (p = 0.04)^6^. To check the application potential of our assay system in clinical samples, we used serum obtained from patients with colorectal cancer (number of samples, n = 5) and healthy individuals (n = 2). DPV measurements were carried out after the incubation of the serum sample on the biotinylated FAM134B antibody-functionalized electrode. A washing step with the PBS (pH 10 mM, pH 7.4) was performed in between steps. As can be seen in Figs 6A and S7, our method effectively detects FAM134B protein from all tested clinical samples with a %RSD of less than 10%. Importantly, DPV voltammograms collected from serum samples are very similar to the ones collected from cell lysates or spiked PBS samples. A similar level of DPV voltammograms from the biological samples have previously been reported^20, 21, 25^. As evident from the results, the expression of FAM134B protein is downregulated in cancer samples when compared to that of normal healthy individuals (Fig. 6A). We found reduced relative current response (less FAM134B protein on the electrode surface) with cancer serum samples compared to that of healthy normal samples (Fig. 6A). We also analyzed the clinical samples with ELISA and noted that results were similar to our assay method (Fig. 6B). These results validate the applicability of the proposed method in clinical sample analysis. The findings supported our previous data, which further indicated the functionality and applicability of the assay system for clinical samples analysis.

In this study, we developed an electrochemical method for detection of FAM134B protein which has several advantages over the conventional methods. Unlike conventional sandwich assays based on primary and secondary antibodies and an electroactive compound to achieve electrochemical detection^24, 32, 33^, the current approach uses a primary antibody and a redox probe, [Fe(CN)_6_]^3−/4−^, to report on the presence of a protein, thereby simplifies the sandwich assay protocol by avoiding the use of a secondary antibody step. The analytical performance (*e.g.* specificity, sensitivity, and reproducibility) of our method is quite comparable with previously reported electrochemical methods for protein detection using the [Fe(CN)_6_]^3−/4−^ redox probe^20, 21, 25, 34^. Additionally, our assay uses only 7 μL of samples and generates electrochemical signal within 1 h and 10 min whereas for ELISA, it takes an average of 100 μL of sample and a longer time (e.g., several hours). Notably, our assay does not require any tedious electrode surface functionalization and utilizes cheap and disposable commercially available screen printed electrodes which provides good assay sensitivity, specificity and reproducibility. The use of electrochemical readout and disposable electrodes adds convenience and portability of the detection system at low cost. The assay has a straightforward design, with readout being performed in two steps, involving the introduction of a non-covalently attached redox system. As FAM134B expression was correlated with cancer stages, pathogenesis and clinicopathological parameters of patients suffering from colorectal cancer, our method may help in easy and cost effective detection of FAM134B expression in cancer patients’ clinical samples^5–8, 11^. To achieve these, further studies with a larger series of clinical cohorts are needed for the functional validation of our current approach.

## Conclusions

In this proof-of-concept study, we have developed a simple and low-cost yet sensitive and specific electrochemical method for the quantitative detection of FAM134B protein in colorectal cancer. This study represents the first demonstration of a label-free electrochemical method for the detection of FAM134B protein using extravidin-coated screen-printed carbon electrode. The level of analytical performance of our method (*i.e.*, reproducibility, % RSD = 7.13), detection limit (10 pg μL^−1^), dynamic range (0.01–100 ng μL^−1^), etc.), may attribute this assay system for the analysis of FAM134B antigen in human diseases. We therefore believe that our assay can be used as a simple and inexpensive alternative to current standard methods, and address the growing demand for inexpensive, stable, simple and sensitive detection of FAM134B protein biomarkers in colon cancer. In addition, the principle of this assay is not limited to only FAM134B proteins, a large number of assays can be fabricated by changing the specific antibodies in the bio-recognition step. Thus, it offers a new avenue for the detection of a wide range of protein-based biomarkers in clinical samples.

## Materials and Methods

### Reagents

Unless otherwise stated, all the reagents and chemicals used in this study are of analytical grade and purchased from Sigma-Aldrich (Sydney, NSW, Australia). FAM134B protein was procured from Santa Cruz biotechnology (Dallas, Texas, USA). Anti-rabbit monoclonal FAM134B antibody and biotin conjugation kit were purchased from Abcam (Melbourne, VIC, Australia). Reagents and washing solutions used in the experiments were prepared with phosphate buffer saline (PBS, 10 mM, pH 7.4). Antibodies, proteins and cell extracts were diluted in phosphate buffered saline (PBS).

### Biotinylation of FAM134B antibody

FAM134B monoclonal antibody were biotinylated using biotin conjugation kit (Abcam) followed by manufacturer's guidelines. Briefly, 10 μL modifier reagent was added to the 100 μL of FAM134B antibody (0.1 mg mL^−1^) and were mixed gently. Then the antibody-modifier mixture was added directly onto the lyophilized biotin (Type B) material and resuspended several times by pipetting. The complex was then kept overnight in the dark at room temperature to conjugate the antibody with biotin. After the incubation, 10 μL of quencher reagent was added to the solution and were mixed gently. Finally, after 30 min incubation at room temperature, the conjugated antibody was stored at −20 °C and used in this study.

### Cell culture and protein extraction

Whole cell proteins from three colon cancer cell lines (SW-480, SW-48 and HCT116) and one non-cancer colon epithelial cell line (FHC) were used in this study. All the cell lines purchased from ATCC (American type culture collection). SW-480 and SW-48 were maintained in RPMI (Roswell Park Memorial Institute) 1640 medium (Life Technologies Australia Pty Ltd, Melbourne, VIC, Australia) containing 10% fetal bovine serum (FBS) (Life Technologies Australia Pty Ltd) and 1% penicillin/streptomycin (Life Technologies Australia Pty Ltd) at 37 °C in a CO_2_ incubator. HCT116 cells were cultured and maintained in DMEM (Dulbecco’s Modified Eagle’s Medium) medium (Life Technologies Australia Pty Ltd) modified with 10% FBS and 1% penicillin/streptomycin at 37 °C in a CO_2_ incubator. FHC cells were cultured in DMEM: F-12 medium (1:1) (Life Technologies Australia Pty Ltd) supplemented with 10 ng mL^−1^ cholera toxin (Sigma-Aldrich), 0.005 mg mL^−1^ insulin (Life Technologies Australia Pty Ltd), 100 ng mL^−1^ hydrocortisone (Sigma-Aldrich), 0.005 mg mL^−1^ transferrin (Life Technologies Australia Pty Ltd) and 10% FBS 37 °C in a CO_2_ incubator. Total proteins were extracted from all cultured cells with lysis buffer (Bio-Rad, Gladesville, NSW, Australia) and subsequently were quantitated by absorbance spectrometry using protein quantification assay kit (Macherey-Nagel, Bethlehem, PA, USA).

### Clinical sample preparation

Serum was separated and collected from peripheral blood samples using standard operating protocols. Briefly, blood was collected in plain tube (no anticoagulant) and left for 30 min to clot. Then the clot was removed by centrifugation (2000 g, 10 min) and the supernatant serum was collected in 1.5 mL tubes and stored at −80 °C. No preservative or protease inhibitors were used in the serum preparation for the current study. The human samples analysis performed with ethic approval from the Human Research Ethic Committee in the Griffith University (MSC/17/10/HREC). Samples were recruited with no selection bias and were carried out in accordance with the approval guidelines and regulations. Also, informed consent was obtained from all subjects included in the present study.

### Electrochemical detection of FAM134B protein in colon cancer

All electrochemical measurements were performed on a CH1040C potentiostat (CH Instruments, Bee Cave, TX, USA) with the three-electrode system printed on a ceramic substrate (length 33 × width 10 × height 0.5) mm (DropSens, Llanera Asturias, Spain). In the three-electrode system, working (diameter = 4 mm), counter and reference electrodes were extravidin/carbon, carbon, and silver-modified electrodes. Initially, the electrodes were incubated with the 7 µL of biotinylated FAM134B antibodies for 20 min followed by the blocking with 7 µL Bovine serum albumin (BSA) (BSA, 0.1%) for another 20 min to minimize the nonspecific adsorption of biomolecules on the extravidin-modified electrodes. Finally, 7 µL of protein samples was incubated for 30 min to form antigen-antibody complex. A washing step with the PBS (pH 10 mM, pH 7.4) was performed in between steps. Differential pulse voltammetric measurements were carried out in PBS (pH 7.4) solution containing 2.5 mM [K_3_Fe(CN)_6_] and 2.5 mM [K_4_Fe(CN)_6_] electrolyte system. DPV signals were recorded from −0.1–0.5 V with a pulse amplitude of 50 mV and width of 50 ms. The relative current changes corresponding to FAM134B antigen binding to the antibody/BSA-bound surface was estimated using eqn (1):

1 $$ \% {i}_{r,FAM{134}Bantigen}=\frac{{i}_{{baseline}-}{i}_{{antibody}/BSA/{antigen}}}{{i}_{{baseline}}}\times 100$$

where *i*_*baseline*_ and *i*_*antibody/BSA/antigen*_ are the mean DPV currents obtained at unmodified (bare) and antibody/BSA/FAM134B antigen-modified electrodes respectively. The difference in relative DPV signals between antibody/BSA- and antibody/BSA/antigen-modified electrodes was estimated using eqn (2):

2 $${\rm{\Delta }}{i}_{{\rm{r}}}= \% {i}_{r,FAM{134}Bantigen}\mbox{--} \% {i}_{r,antibody/BSA}$$

where %*i*_*r*, *antibody/BSA*_ is the relative current change corresponding to anti-FAM134B/BSA binding to the extravidin-modified screen-printed electrode and estimated using eqn (3):

3 $$ \% {i}_{r,{antibody}/BSA}=\frac{{i}_{{baseline}-}{i}_{{antibody}/BSA}}{{i}_{{baseline}}}\times 100$$

### Enzyme-linked immunosorbent assay

Biotinylated FAM134B antibody diluted (10 μg mL^−1^) in PBS was added to the pierce neutravidin coated 96-well plate (Thermo Fisher Scientific, VIC, Australia) and incubated overnight at 4 °C. After being washed with PBS, 5% BSA in PBS was added to the wells and incubated for 1 h at room temperature for blocking. FAM134B protein/cell extracts/serum samples were then applied to the plate and incubated for 1 h at room temperature. FAM134B primary antibody (20 μg mL^−1^) was added to each well after a PBS wash. Corresponding HRP (horseradish peroxidase)-conjugated secondary antibody was then added to the plate. Subsequently, substrate for enzymatic reaction was added and incubated 1 h. Finally, followed by washing with PBS, absorbance was taken with microplate reader.

### Statistical analysis

All the experimental data were computerized and statistical analysis was performed using the Statistical Package for Social Sciences for Windows (version 22.0, IBM SPSS Inc., New York, USA). Independent t-test and ANOVA were performed for the analysis of continuous variables in categories. Significance level of the tests was taken at p < 0.05.

## Electronic supplementary material

SI-RE-revision
