# Supplementary material for: An electrochemical method for sensitive and rapid detection of FAM134B protein in colon cancer samples
Source: Sci Rep. 2017 Mar 9;7:133. doi: 10.1038/s41598-017-00206-8 (PMC5428029; doi:10.1038/s41598-017-00206-8)
Supplement: Supplementary file 1 — SI-RE-revision [file 41598_2017_206_MOESM1_ESM.pdf]

Supplementary information

for

An electrochemical method for sensitive and rapid detection of FAM134B protein in colon cancer samples

Farhadul Islam,[a] Md. Hakimul Haque,[ab] Sharda Yadav[bc], Md Nazmul Islam[bc],Vinod Gopalan,[a]* Nam-Trung Nguyen,[c] Alfred K. Lam,[a]* Muhammad J. A. Shiddiky [b,c]*

Western blot analysis

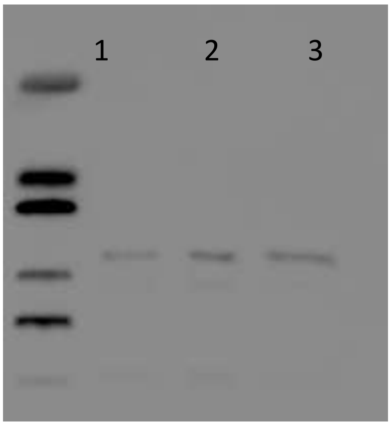

Figure S1. Western blot analysis was performed to identify the specificity of FAM134B antibody (abcam) in colon cancer (SW480; lane 3, SW48; lane 2 and HCT116; lane 1) cell extracts. Total proteins from these cells were extracted with a lysis buffer (Bio-Rad, Gladesville, NSW, Australia). Subsequently, the proteins were quantitated by absorbance spectrometry. Afterwards, 30 μg of total protein was separated by 15% SDS-PAGE (Bio-Rad) and transferred to nitrocellulose membranes using Trans-Blot® Turbo transfer system (Bio-Rad). The membrane was blocked with 5% non-fat milk powder for 1 h at room temperature. Then the membrane was incubated with anti-rabbit FAM134B antibody overnight at 4°C. Membranes were then incubated with secondary antibody (Santa Cruz) (1:5000) at room temperature for 2 h. FAM134B protein (~54 KD) band was developed and detected with a chemiluminscence HRP detection kit (Bio-Rad, USA). We noted single band in the all the cell line extracts. The results implied that FAM134B antibody is highly specific for the target.

Optimization of BSA concentration

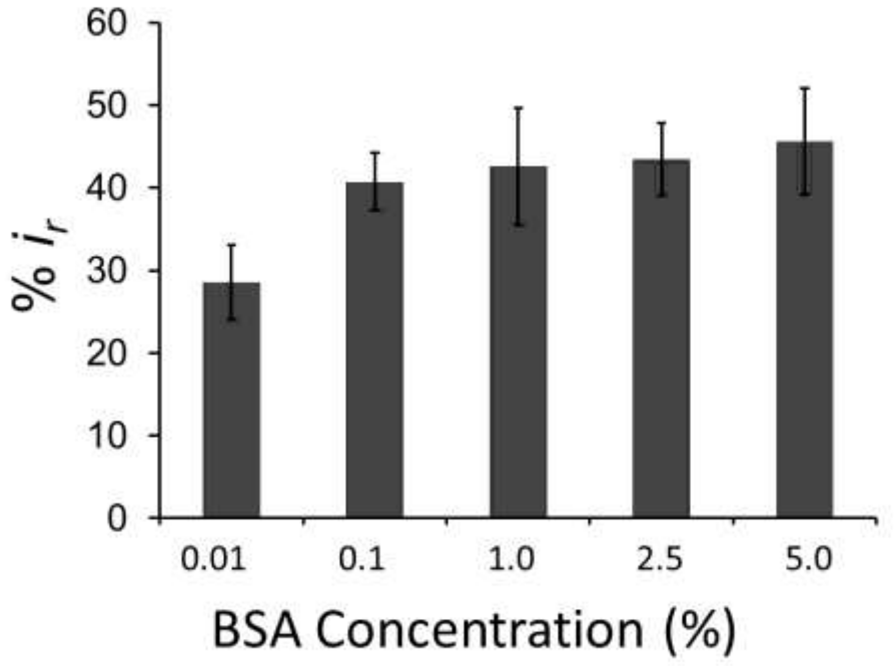

Figure S2. Mean percentages of current changes (%i_r) at different concentration of BSA (incubation time = 20 min).

Immuno-precipitation analysis

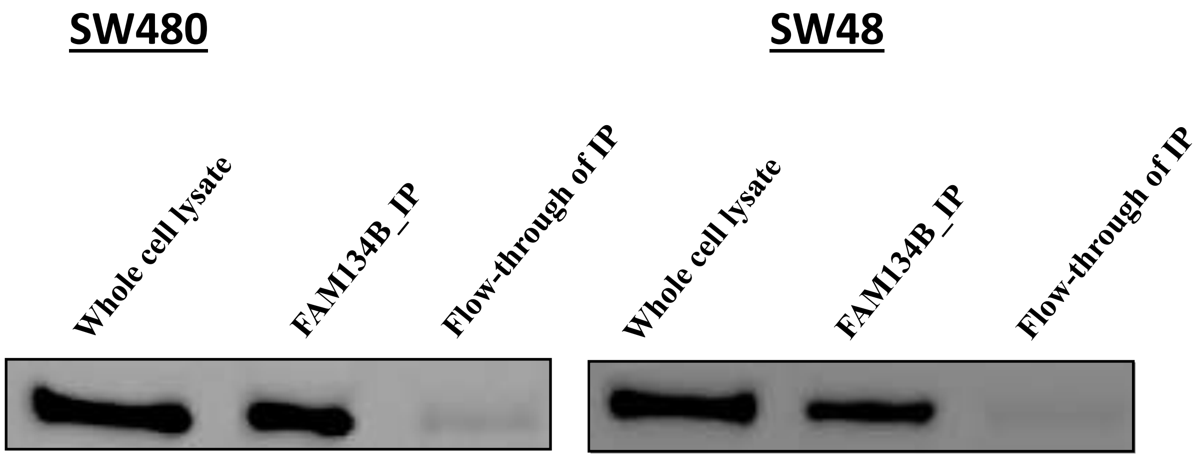

Figure S3. FAM134B protein was immuno-precipitated out from SW480 and SW48 cell extracts using pierce co-immunoprecipitation kit (Thermo Fisher Scientific) according to the manufacturer's guideline. In this study, we used the flow-through wash buffer as FAM134B knockout sample. After capturing of FAM134B protein in co-immunoprecipitation column, the flow-through washes solution should not contain any FAM134B protein. We checked the absence of FAM134B protein in flow-through wash solutions by SDS-PAGE followed by Western blot analysis. We used whole cell lysate and IP sample to compare the presence of FAM134B protein in the flow-through sample. We observed a huge intense proteins band in the whole cell lysate and FAM134B-IP lane, whereas in the flow-through lane we did not find any band.

Electrochemical assay

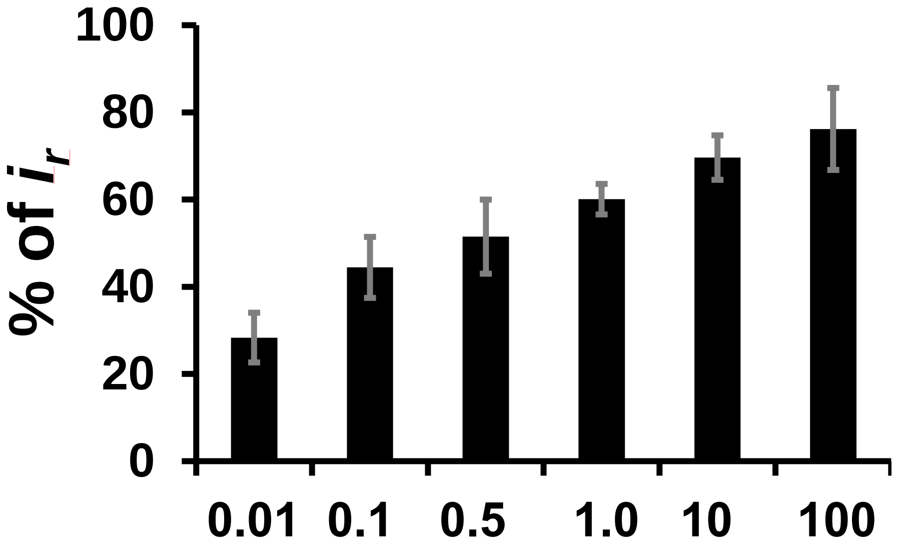

Figure S4. A linear increase of % i_r was observed with a serial increment of FAM134B protein concentration within the dynamic range of 0.01 ng μL^{-1} to 100 ng μL^{-1}. Each bar graph represents the average of the three separate trials (n = 3) and error bars represents the standard deviation of minimum of 3 experimental replicates.

ELISA

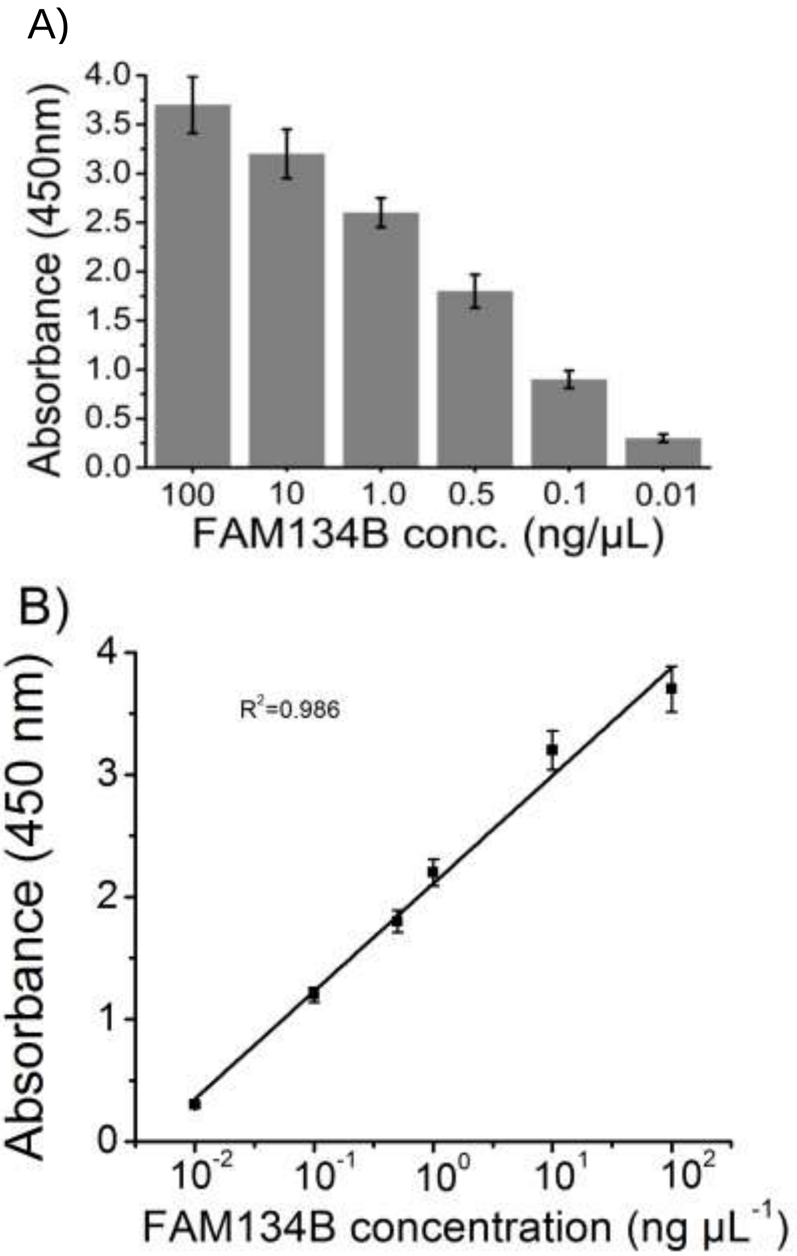

Figure S5. A numbers of dilutions (0.01 to 100 ng μL^{-1}) of FAM134B protein were prepared in PBS and absorbance (optical density) was taken at 450nm followed by ELISA protocol. Linearly increasing absorbance was noted with the increasing concentration of protein (A). A standard curve was then constructed using optical density with a correlation co-efficient of 0.986 (B). Each bar graph represents the average of the three separate trials (n = 3) and error bars represents the standard deviation of minimum of 3 experimental replicates.

Analysis of cell extracts

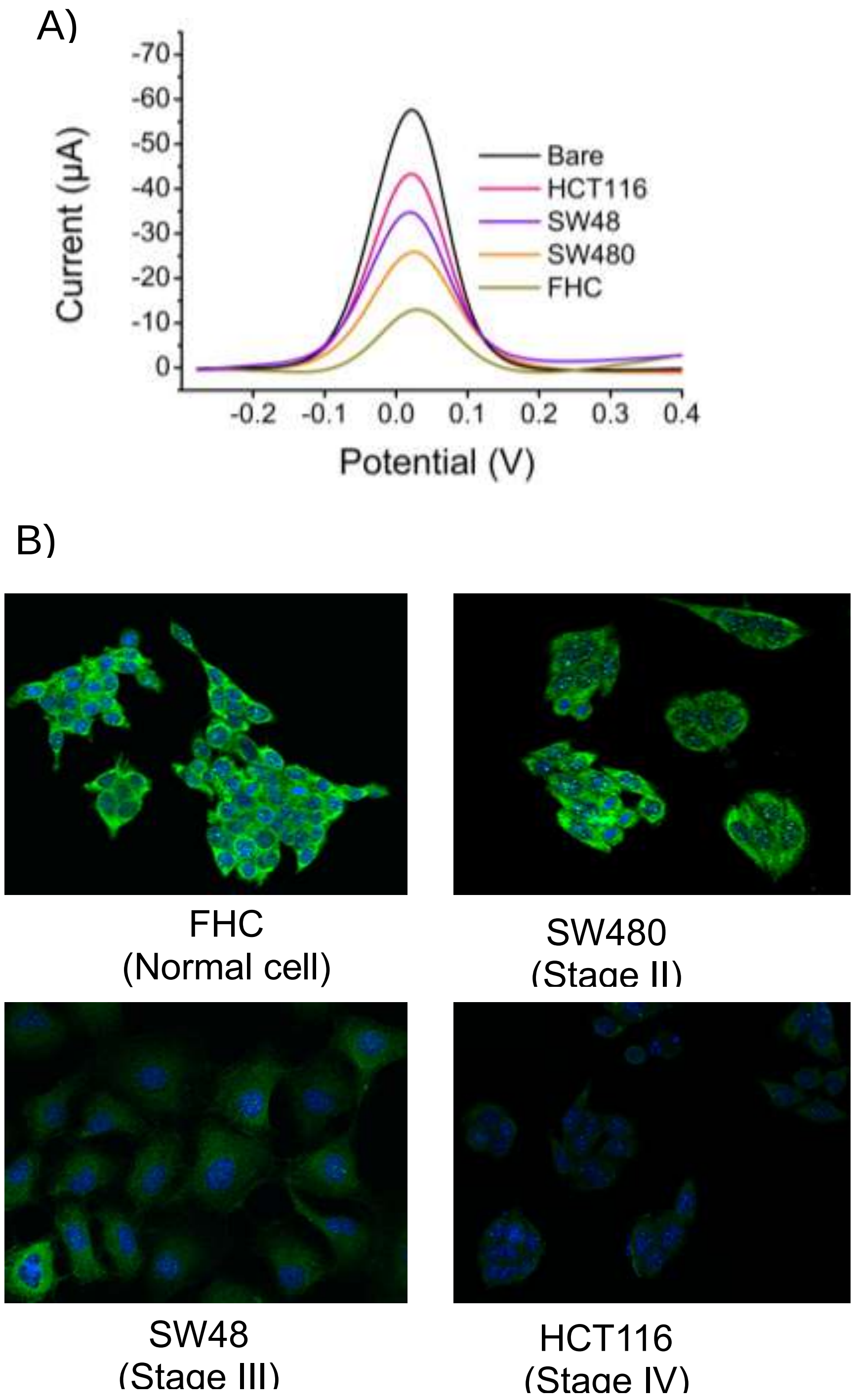

Figure S6. (A) DPV current signals obtained from different cells extracts are significantly different. Normal colon epithelial cells (FHC) exhibited maximum reduction (% i_r = 108.79 ± 8.7%) of current signals compared to that of bare electrode. SW480, stage II colon cancer cell showed % i_r = 63.92 ± 9 reduction of DPV signals. Similarly stage III (SW48) and stage

IV (HCT116) colon cancer cells generated less relative current signals compared to that of normal cell (FHC). (**B)** Stage dependent expression of FAM134B protein further confirmed with immunofluorescence microscopy. We noted strong green signal (expression of FAM134B protein) in FHC cells whereas the green signal became weak with the progression of disease stages and the trend of green signal is FHC>SW480>SW48>HCT116.

Analysis of clinical samples

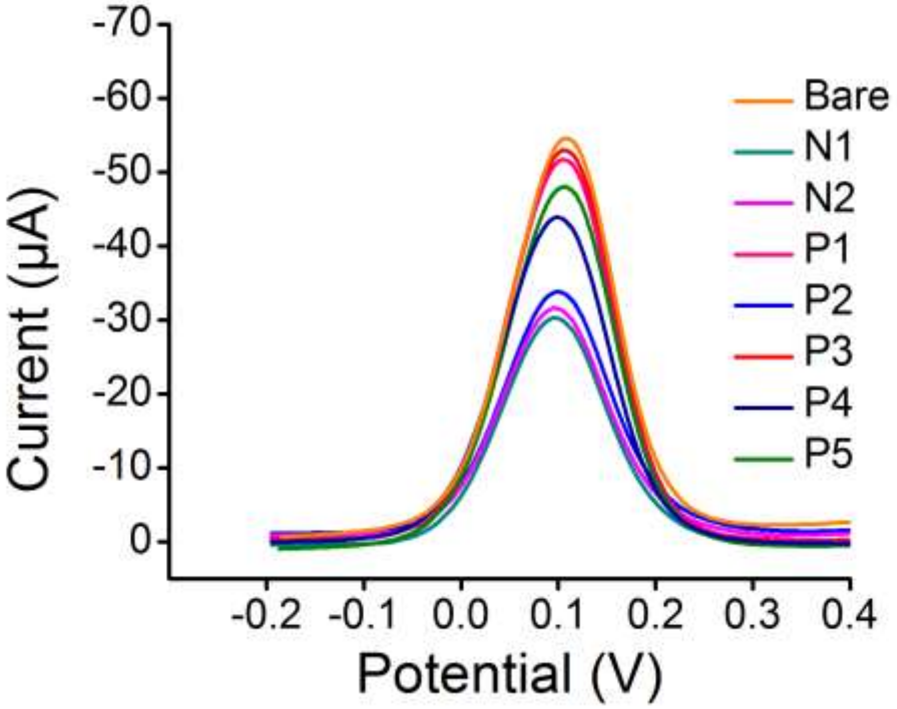

Figure S7. DPV current signal for colorectal cancer samples (P1 to P5) and healthy individuals (N1 and N2).
